# Influencing factors of medication literacy among community-dwelling older adult patients with hypertension: a study based on social learning theory

**DOI:** 10.3389/fphar.2023.1184701

**Published:** 2023-06-02

**Authors:** Tingting Lu, Zhen Yang, Ping Chen, Jingyan Li, Chen Zheng, Linghui Kong, Huijun Zhang

**Affiliations:** ^1^ Department of Nursing, Jinzhou Medical University, Jinzhou, China; ^2^ Department of Nursing, China Medical University, Shenyang, China

**Keywords:** older adult, hypertension, medication literacy, influencing factors, structural equation model

## Abstract

**Objective:** This study aimed to examine the factors affecting medication literacy in community-dwelling older adults with hypertension, guided by social learning theory. It sought to analyze the pathways these factors influenced and provide a theoretical foundation for designing targeted intervention programs.

**Study design:** This is a cross-sectional study.

**Methods:** From October 2022 to February 2023, a total of 432 community-dwelling older adults with hypertension from Linghe District, Guta District, and Taihe District, Jinzhou City, Liaoning Province, China, were selected using convenience sampling. Data were collected using a socio-demographic questionnaire, a medication literacy questionnaire, the Brief Illness Perception Questionnaire, the General Self-efficacy Scale, and the Perceived Social Support Scale. The collected data were analyzed with Kruskal-Wallis and Mann-Whitney tests, correlation analysis, multiple stepwise regression analysis, and structural equation modeling (SEM).

**Results:** The medication literacy score among the participants was 3.83 ± 1.91. Multi-factor analysis revealed key factors affecting their medication literacy, including blood pressure-control status, utilization of community health education resources, receiving guidance for medication usage, marital status, number of annual visits, social support, self-efficacy, and disease perception. The SEM based on social learning theory showed that general self-efficacy mediated the relationship between social support, disease perception, and medication literacy.

**Conclusion:** The present study developed a model and provided potential intervention strategies to improve medication literacy, knowledge, and safety among community-dwelling older adults with hypertension, considering the relationships between the identified variables.

## Introduction

An aging population has led to a rise in older adults with chronic diseases like hypertension, which affects health and quality of life due to its associated risks. In China, 59.4% of older adults suffered from high blood pressure (Li et al., 2018). However, although the prevalence of hypertension is high in older adults, awareness, treatment, and control rates of the disease are relatively low (Wang, Chen, Zhang). Pharmacological treatments are essential for controlling hypertension progression. Studies have shown that good medication compliance can effectively manage blood pressure and improve overall management quality (Lochner et al., 2012). However, older adults, as a vulnerable group, often have limited understanding and capacity to safely use medications (Gavrilova et al., 2019; Marseille et al., 2021). A correct understanding of medication knowledge will strengthen medication compliance and the ability to safely administer drugs in older adult patients with hypertension (Abu et al., 2018; Shi et al., 2019). Therefore, strengthening older adults’ comprehension and processing of medication information is crucial for minimizing irrational drug use, enhancing medication safety, and reducing complications.

The concept of medication literacy was proposed on the basis of health literacy (Sørensen et al., 2012). Pouliot et al. defined medication literacy as an individual’s ability to obtain, understand, communicate, calculate, and process information about a specific drug (Pouliot et al., 2018). Studies have shown that low medication compliance and irrational medication in older hypertensive patients have become important factors affecting the poor blood pressure control effect. Relevant evidence showed that patients’ medication literacy can improve their ability to use drugs safely, correctly, and rationally (Ngoh, 2003; Yazicioglu and Yardan, 2021). In addition, Son KJ et al. pointed out that the chronic disease management plan is a community-based intervention, community education and the popularization of medication knowledge can enhance the understanding of hypertension patients the disease, and so as to better control the disease (Calano et al., 2019; Son et al., 2019). Therefore, it is necessary to pay attention to the medication literacy of community-dwelling older adult patients with hypertension.

Social learning theory was developed by Bandura. He proposed the ternary interaction theory and self-efficacy theory (Bahn, 2001). In ternary interaction theory, Bandura believed that the environment, cognition, and behavior are whole, and these three factors and their interactions affect behavior. He believed that behavior itself is a byproduct of the interaction between individual cognition and environment. In self-efficacy theory, he explained the concept of self-efficacy, which he believed would have an impact on individual behavior (Bahn, 2001; Calano et al., 2019). In this study, cognition was defined as the patient’s perception of the disease, the environment was defined as the degree of external support received by the patient, and behavior was defined as the process by which the patient self-learns medication knowledge and makes correct medication decisions (medication literacy). Based on the relationship between cognition, environment, behavior, and self-efficacy proposed by social learning theory, this study constructed a SEM of disease perception, social support, self-efficacy, and medication literacy in older adult patients with hypertension.

Existing studies on patients’ medication literacy are often limited to specific clinical diseases, such as coronary heart disease or renal failure (Rosenstock et al., 1988; Wang, 2011), and there are fewer studies on medication literacy in the old adults. For older adult patients with hypertensionwho need to take antihypertensive drugs for a long time, improving their drug literacy is of great significance for strengthening disease management and reducing the occurrence of complications (Zhong et al., 2020). Therefore, understanding the factors affecting the medication literacy of elderly hypertension patients is of certain significance for the formulation of appropriate intervention measures. This will help improve the quality of hypertension management and the quality of life of patients in their later years.

## Materials and methods

### Study design and participants

This is a cross-sectional study. The inclusion criteria for participants were:

1. Diagnosed with essential hypertension meeting the diagnostic criteria of the Chinese Guidelines for Hypertension Prevention and Treatment (2018 Revised Edition) (Chinese Hypertension Prevention and Treatment Guidelines Revision Committee, 2019).
2. At least 2 weeks of prescribed hypertension medication.
3. Participants aged 60 years or older.
4. Resided in the community for a minimum of 1 year.
5. Exhibited clear awareness and normal communication skills.
6. Volunteered to participate in the study.

The exclusion criteria were:

1. Patients with secondary hypertension.
2. Patients with severe cardiovascular, brain, kidney, liver, or other diseases.
3. Individuals with mental illness.

This study employed social learning theory to construct a SEM that analyzed the action paths of each variable. SEM analysis requires a large sample size (Jak and Cheung, 2020). According to the sample size requirements of Kendall multivariate analysis, the sample size of the influencing factor study is at least 5–10 times the number of variables (Kendall, 1975). A total of 24 variables were included in this study, which was calculated as 10 times the number of variables, and a 20% loss rate was taken into account, resulting in a sample size of at least 288 cases. In order to ensure the reliability of the results, a total of 432 older adult patients with hypertension were recruited.

### Variables and measurements

The study utilized a socio-demography questionnaire, medication literacy questionnaire, Brief Illness Perception Questionnaire (BIPQ), Perceived Social Support Scale (PSSS), and General Self-Efficacy Scale (GSES) as research tools.

### Socio-demography questionnaire

The socio-demography questionnaire collected information on various factors, such as age, gender, education level, occupational status, marital status, years with hypertension, years taking medication, personal financial status, personal health status, number of annual visits, guidance when taking drugs, blood pressure control status, multiple disease coexistence, medication side effects, the establishment of health records in the community, and utilization of community education resources.

### Medication literacy questionnaire

The medication literacy questionnaire utilized the scale developed by Maniaci et al. (Maniaci et al., 2008) and culturally adapted by Zheng Feng et al., with a Cronbach’s *α* coefficient of 0.850 (Zheng et al., 2020). There are 9 items in this scale, since the study population of this study were community-dwelling older adult patients with hypertension, item 1 was deleted: May I ask whether you have been discharged with medicine this time? Items 7 and 9 were not scored as there are no correct answers. The total score was 6 points, with higher scores indicating higher medication literacy. A score of 0–2 indicates that the level of medication literacy is at a low level, a score of three to four indicates a medium level, and a score of five to six indicates a high level of medication literacy.

### Brief illness perception questionnaire (BIPQ)

BIPQ was developed by Broadbent et al. (Broadbent et al., 2015). Mei Ya-qi et al. translated it into Chinese. The scale’s Cronbach’s *α* coefficient was 0.77, and its split-half reliability was 0.81 (Mei et al., 2015). The questionnaire included three dimensions: disease cognition, emotion and disease understanding. There are 9 items in this questionnaire, among which the ninth open question does not count. The scale ranged from 0 to 80 points. Higher scores indicated more negative disease perceptions and emotions.

### Perceived social support scale (PSSS)

PSSS was developed by Zimet et al. (Zimet et al., 1988). Zhang Fan et al. translated the PSSS into Chinese. The Cronbach’s *α* coefficient of this scale was 0.840 and the retest reliability was 0.791 (Zhang et al., 2018). It consisted 12 items in three dimensions: family support, friend support and other support. The scale scores ranged from 12 to 84, with higher scores indicating greater perceived social support.

### General self-efficacy scale (GSES)

The GSES scale was developed by Schwarzer (Schwarzer and Born, 1997). Wang Cai-kang et al. introduced it into China, and the reliability of the scale after the translation was 0.87 (Wang et al., 2001). The scale has a total of 10 items with scores ranging from 10 to 40. Higher scores indicated greater self-efficacy.

### Data collection

Data was collected between October 2022 and February 2023 in Linghe District, Guta District, and Taihe District, Jinzhou City. Researchers visited community senior centers and after undergoing training, explained the study purpose, content, and processes to eligible older adults. Informed consent was obtained, and the research questionnaires were administered. For participants with literacy limitations, researchers read the questionnaires and recorded responses. The completed questionnaires were checked for validity, and a total of 450 were distributed. Of those, 432 valid questionnaires were returned, with an effective recovery rate of 96%. To prevent data entry errors, the collected data was reviewed by two people and entered into Excel 2016.

### Statistical analysis

Data were analyzed using SPSS25.0 software. Descriptive statistics were conducted; measurement data were described as means and standard deviations, while count data were described using frequency and composition ratio. Kruskal-Wallis and Mann-Whitney tests analyzed the impact of different socio-demographic characteristics on medication literacy. Multiple linear regression was used to analyze key factors affecting medication literacy, and Spearman correlation coefficients analyzed the correlation between variables. The SEM was constructed using Amos23.0 software, employing the maximum likelihood method for parameter estimation. A *p*-value <0.05 indicated statistical significance.

## Results

### Demographic characteristics

A total of 432 valid questionnaires were included in the study, with an average age of 70.70 ± 6.83 years. Males accounted for 49.1% and females for 50.9%. Of the participants, 300 were retired, 210 had elementary school education or below, and 267 were married. Additional socio-demographic data are presented in Table 1.

**TABLE 1:** The influence of different Social demography characteristics on medication literacy (means ± SD, score) (*n* = 432).

Variables	*n* (%)	Score	Statistical value	*p*-Value
Age			χ^2^ = 22.428	<0.001
60–70	220 (50.9)	4.21 ± 1.83		
71–80	169 (39.1)	3.53 ± 1.86		
>80	43 (10.0)	3.02 ± 2.16		
Gender			Z = −1.294	0.196
male	212 (49.1)	3.89 ± 2.01		
Female	220 (50.9)	3.76 ± 1.82		
Educational level			χ^2^ = 1.959	0.581
Primary and below	210 (48.6)	3.73 ± 1.98		
Middle and high school	144 (33.3)	3.89 ± 1.78		
Secondary and junior colleges	69 (16.0)	3.97 ± 1.87		
Bachelor degree or above	9 (2.1)	4.00 ± 2.78		
Occupational status			χ^2^ = 4.211	0.122
Working	33 (7.6)	4.52 ± 1.35		
unemployed	99 (22.9)	3.88 ± 2.03		
Retired	300 (69.4)	3.73 ± 1.92		
Marital status			χ^2^ = 43.921	<0.001
Married	267 (61.8)	4.32 ± 1.64		
Divorced	49 (11.3)	3.18 ± 2.14		
Widowed	105 (24.3)	3.01 ± 2.04		
Unmarried	11 (2.5)	2.45 ± 1.86		
Years with hypertension			χ^2^ = 0.083	0.960
<5 years	62 (14.4)	3.71 ± 2.11		
5–10 years	169 (39.1)	3.86 ± 1.85		
>10 years	201 (46.5)	3.83 ± 1.92		
Years taking medication			χ^2^ = 0.950	0.622
<5 years	100 (23.1)	3.58 ± 2.15		
5–10 years	197 (45.6)	3.91 ± 1.86		
>10 years	135 (31.3)	3.89 ± 1.80		
Personal financial status			χ^2^ = 17.011	<0.001
Dissatisfied	126 (29.2)	3.39 ± 2.12		
General	185 (42.8)	4.28 ± 1.69		
Satisfactory	121 (28.0)	3.59 ± 1.88		
Personal health status			χ^2^ = 7.048	0.029
Dissatisfied	181 (41.9)	3.54 ± 2.07		
General	161 (37.3)	4.16 ± 1.72		
Satisfactory	90 (20.8)	3.81 ± 1.84		
Number of annual visits			χ^2^ = 49.602	<0.001
None	68 (15.7)	2.57 ± 2.06		
1 time	123 (28.5)	3.53 ± 1.79		
2 times	106 (24.5)	4.30 ± 1.69		
3times or more	135 (31.3)	4.36 ± 1.78		
Guidance when taking drugs			Z = −9.445	<0.001
Yes	258 (59.7)	4.56 ± 1.50		
No	174 (40.3)	2.74 ± 1.95		
Blood pressure control status			Z = −10.584	<0.001
Normal	275 (63.7)	4.61 ± 1.40		
Abnormal	157 (36.3)	2.46 ± 1.92		
Multiple diseases coexistence			Z = −0.149	0.882
Yes	210 (48.6)	3.78 ± 1.99		
No	222 (51.4)	3.87 ± 1.85		
Medication side effects			Z = −1.541	0.123
Yes	179 (41.4)	4.00 ± 1.85		
No	253 (58.6)	3.70 ± 1.95		
Establish community health records			Z = −8.222	<0.001
Yes	192 (44.4)	4.66 ± 1.49		
No	240 (55.6)	3.16 ± 1.96		
Use of community health education resources			Z = −10.740	<0.001
Yes	197 (45.6)	4.87 ± 1.32		
No	235 (54.4)	2.95 ± 1.89		

### Influence of different social demographic data on medication literacy of community-dwelling older adult patients with hypertension

The score of the dependent variable medication literacy was an integer between 0 and 6, which was a discrete variable and did not follow a normal distribution. Therefore, Mann-Whitney (dichotomous variables) or Kruskal-Wallis tests (multi-categorical variables) were used to compare the effect of different sociodemographic factors on medication literacy (*p* < 0.05). The results revealed statistically significant differences for age, marital status, personal financial status, personal health status, number of visits per year, blood pressure control status, guidance during medication usage, community health record establishment, and community education resource utilization. Specific values are shown in Table 1.

### Scores of various scales in community-dwelling older adult patients with hypertension

Scores for each scale from 432 older adults with hypertension were analyzed. Descriptive statistics showed a medication literacy score of 3.83 ± 1.91, a BIPQ score of 38.54 ± 16.47, a PSSS score of 51.29 ± 15.76, and a GSES score of 25.78 ± 6.70. The scores of disease cognition, emotion and disease understanding in the BIPQ were 23.93 ± 10.71, 9.96 ± 4.60, and 4.65 ± 2.68, respectively. The scores of family support, friend support, and other support in the PSSS were 17.33 ± 5.58, 16.62 ± 5.50, and 17.34 ± 5.56, respectively (Table 2).

**TABLE 2:** The score of each scale (*n* = 432).

Scale	dimension	Score
Medication literacy questionnaire	Total score	3.83 ± 1.91
Perceived social support scale	Total score	51.29 ± 15.76
	Family support	17.33 ± 5.58
	Friend support	16.62 ± 5.50
	Other support	17.34 ± 5.56
General self-efficacy scale	Total score	25.78 ± 6.70
Brief illness perception questionnaire	Total score	38.54 ± 16.47
	Disease cognition	23.93 ± 10.71
	Emotion	9.96 ± 4.60
	Disease understanding	4.65 ± 2.68

### Multi-factor analysis of influencing factors of medication literacy

Statistically significant sociodemographic variables were used in a multiple stepwise linear regression analysis to explore key factors influencing medication literacy. The multi-factor analysis results revealed that blood pressure control status, utilization of community education resources, guidance during medication usage, marital status, number of annual visits, social support, self-efficacy, and disease perception significantly influenced medication literacy (*p* < 0.05), explaining 63.5% of the variation (*R* ^2^ = 63.5%). The specific results are shown in Table 3. The results showed that medication literacy in patients with good blood pressure control was higher than that in patients with poor blood pressure control, patients who were good at using community educational resources had higher medication literacy than those who did not, the medication literacy of patients with guidance was higher than that of patients without guidance, married patients had higher levels of medication knowledge than those in other forms of marriage, the more visits a year a patient had, the higher their medication literacy. In addition, the higher the social support patients received, the better their medication literacy, when patients perceive less negative emotions of disease, their medication literacy will also be improved, the higher the self-efficacy of patients, the higher the medication literacy.

**TABLE 3:** Influence of different independent variables on medication literacy (*n* = 432).

Independent variable	Regression coefficient (B)	Standard error (SE)	Standardized regression coefficient (β)	t	*p*
Constant	4.351	0.442	—	9.836	<0.001
Control of blood pressure status	−0.860	0.139	−0.216	−6.182	<0.001
Social support	0.035	0.004	0.289	8.563	<0.001
Use of community health education resources	−0.549	0.140	0.143	−3.929	<0.001
Guidance when taking medication	−0.870	0.141	−0.223	−6.174	<0.001
Self-efficacy	0.040	0.010	0.140	3.945	<0.001
Number of visits in a year	0.258	0.055	0.144	4.704	<0.001
Marital status	−0.230	0.066	−0.111	−3.487	0.001
Disease perception	−0.011	0.004	−0.096	−2.724	0.007

### Correlation between variables

A positive correlation existed between social support, self-efficacy, and medication literacy, while disease perception was negatively correlated with medication literacy. The correlation between variables is presented in Table 4.

**TABLE 4:** The correlation between variables.

Variables	Medication literacy	Disease perception	Social support	Self efficacy
	R P	R P	R P	R P
Medication literacy	1.000			
Disease perception	−0.465 < 0.001	1.000		
Social support	0.449 < 0.001	−0.210 < 0.001	1.000	
Self efficacy	0.431 < 0.001	−0.382 < 0.001	0.415 < 0.001	1.000

### SME of factors influencing medication literacy in community-dwelling older adult patients with hypertension

#### Initial model assumption

**FIGURE 1:**
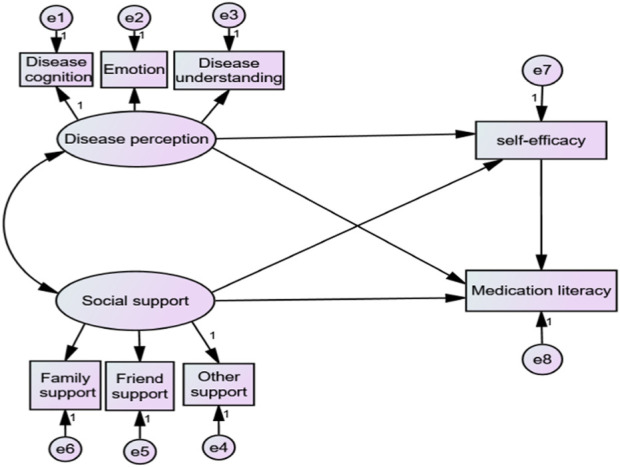
Initial model assumption.

Based on social learning theory, researchers made the following assumptions about the model. In social learning theory, cognition and environment have a direct influence on behavior, and self-efficacy as a sense of belief can also have a certain effect on behavior. Therefore, it is assumed that disease perception and social support can directly affect medication literacy, and both can influence medication literacy through self-efficacy. As shown in Figure 1, the perceived social support scale and brief illness perception questionnaire include three dimensions (Zimet et al., 1988; Broadbent et al., 2015), while the general self-efficacy scale and medication literacy questionnaire are unidimensional (Schwarzer and Born, 1997; Maniaci et al., 2008). So we replaced latent variables with observational variables for self-efficacy and medication literacy. In the hypothesis model, social support and disease perception served as independent variables, self-efficacy as a mediating variable, and medication literacy as a dependent variable.

#### Model fitting

**FIGURE 2:**
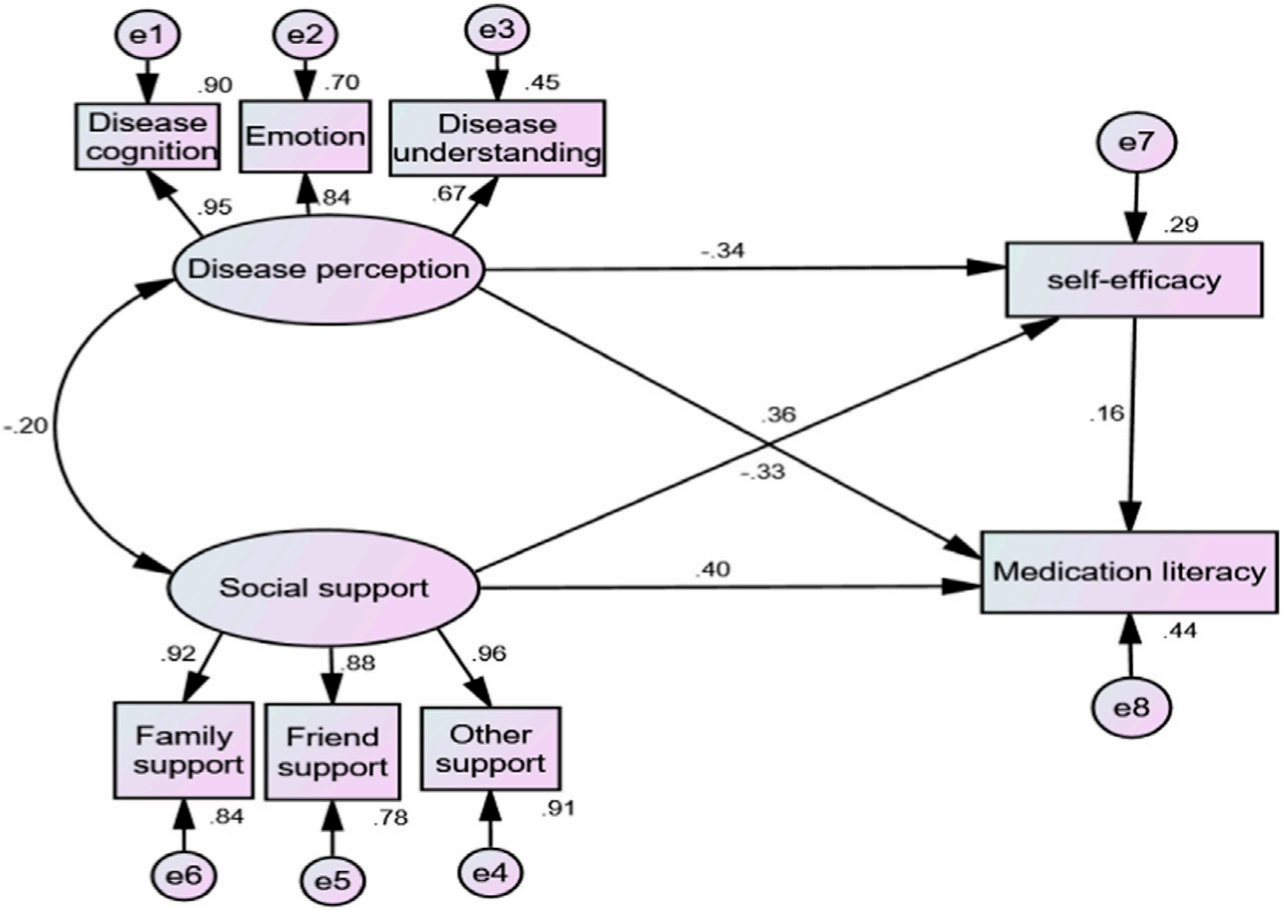
Final model path and standardized regression coefficients.

To confirm the model’s validity, researchers evaluated the model’s degree of fit. Table 5 displays the results, which demonstrate a satisfactory fit. All path coefficients were statistically significant (*p* < 0.01). Furthermore, the effects of disease perception, social support, and self-efficacy on medication literacy were −0.33, 0.16, and 0.40, respectively. The final model is illustrated in Figure 2. In the SEM, the indirect effect of self-efficacy on social support and medication literacy was 0.056, while the indirect effect on disease perception and medication literacy was −0.053. The 95% confidence intervals, tested using the Bootstrap method, were (0.022,0.099) and (−0.091,−0.023), respectively. The absence of 0 signifies the statistical significance of the indirect effect. Consequently, self-efficacy played a partial mediating role between social support and medication literacy and between disease perception and medication literacy.

**TABLE 5:** Model fitting table.

Index	χ2	χ2/df	GFI	NFI	IFI	CFI	RMSEA
Model index	40.839	2.552	0.977	0.982	0.989	0.989	0.060
References index		<3	>0.90	>0.90	>0.90	>0.90	<0.08

## Discussion

### The medication literacy of community-dwelling older adult patients with hypertension needs to be improved

While medication adherence among older adult patients with hypertension in communities has been a research focus, there has been little attention on enhancing their medication literacy. Understanding medication information can aid older adult patients with hypertension in rationalizing their medication behaviors (Ma et al., 2020). This study discovered that the medication literacy score for community-dwelling elderly hypertension patients was 3.83 ± 1.91, which is moderate. This finding is consistent with Zhang Kunxiu et al.’s study, which reported that older adult patients with hypertension had a medium medication literacy level but needed improvement due to limited drug use knowledge and unfirm belief in taking medicine (Zhang et al., 2021). In this study, the medication literacy of community-dwelling older adult patients with hypertension was at a medium level, potentially related to the strengthened community health education on chronic diseases. Nonetheless, older adults’ physiological and cognitive functions decline, leading to limited medication use knowledge and susceptibility to the influence of friends, family, hospitals, communities, and other factors. Thus, enhancing the “hospital-community-family” trinity chronic disease management mode is necessary. Kruskal-Wallis analysis results also indicated that older adults’ medication literacy declines with age, emphasizing the need to reinforce the popularization and publicity of drug knowledge for older adults.

Multiple linear regression results revealed that older adult hypertensive patients with regular blood pressure control exhibited higher medication literacy, suggesting that drug knowledge could help control blood pressure to some extent. Older adult patients with hypertension who utilize community education resources also possess higher medication literacy, underlining the positive role communities play in chronic disease management. Therefore, community workers’ professional skills should be cultivated, and the depth and breadth of medication knowledge popularization in communities should be increased. Older adult patients with hypertension who receive guidance, are married, and visit more frequently annually have higher medication literacy, reflecting the importance of family and hospital guidance. Besides fortifying elderly hypertension patients’ understanding of medication knowledge, patients’ families’ medication literacy should also be strengthened. Similarly, medicine knowledge interpretation and communication by doctors and nurses in hospitals are crucial.

### Analysis of the influence of social support on medication literacy

Environmental factors are among the most critical factors in social learning theory. This study found a positive correlation (0.449, *p* < 0.01) between social support and medication literacy. This is aligned with the results of Shen Z et al., which highlighted the important role of social support in promoting medication literacy in older adult patients with hypertension (Shen et al., 2022). In the SEM, the effect coefficient of social support on medication literacy was 0.40, implying the importance of boosting social support to improve older adult patients with hypertension. The support from family, friends, and others for older adult patients with hypertension impacts their medication literacy improvement. This relationship may be associated with medication information and knowledge transmission by family, friends, and other individuals, the establishment of support mechanisms, and the enhancement of understanding medication information during communication, ultimately promoting safe, regular, and reasonable drug use. The external environment’s significance to cognition is also evident. As such, while strengthening patients’ understanding and handling of drug knowledge, educating family members who influence older adult hypertension patients’ medication literacy improvement is equally important.

### Analysis of the influence of disease perception on medication literacy

Social learning theory posits that behavior is an interactional by-product between individual cognition and environment and acknowledges their essential role in individual behavior (Cui et al., 2022). In this study, older adult patients with hypertension had a disease perception score of 38.54 ± 16.47 and a disease cognition score of 23.93 ± 10.71, which indicated that their negative emotions were at a lower-middle level. This may be related to the high degree of social support patients receive. Higher social support results in reduced negative disease cognition. In the SEM, the direct effect of disease perception on medication literacy was −0.33. It suggested that the higher the negative emotion of older adult patients with hypertension, the lower their medication literacy A patient’s perception of his or her illness is a key predictor of his or her behavioral tendencies (Pahria et al., 2022). Negative disease cognition will make patients more inclined to take negative behaviors, weaken patients’ confidence in disease control, reduce the initiative of hypertension patients to learn drug knowledge, and is not conducive to the improvement of medication literacy. Therefore, reducing the negative emotion of older adult patients with hypertension to the disease can improve their bad cognition of the disease and enhance their motivation to learn medication knowledge.

### Analysis of the influence of self-efficacy on medication literacy

Bandura postulated that self-efficacy influences not only individual goal selection but also the manner in which individuals behave (Bahn, 2001). Self-efficacy refers to a person’s estimation and judgment of their ability to accomplish a specific task (Shorey et al., 2021). In this study, the direct effect of self-efficacy on medication literacy was 0.16, indicating that enhancing self-efficacy in older adult patients with hypertension can improve their learning and understanding of medication knowledge. Shen Z et al. found a mediating role of self-efficacy between medication literacy and medication compliance, emphasizing the importance of improving self-efficacy in medication compliance among older adult patients with hypertension (Shen et al., 2020). In this study, self-efficacy mediated social support and medication literacy, as well as disease perception and medication literacy. Strengthening social support can enhance self-efficacy in older adult patients with hypertension, thereby improving their medication literacy levels. Similarly, reducing negative emotions related to the disease can enhance their self-efficacy and subsequently improve medication literacy levels. Positive beliefs can encourage patients to adopt positive behaviors (Khademian et al., 2020). In practice, nursing staff should prioritize psychological counseling for patients and boost their confidence in disease treatment.

### Strategies and suggestions for improving medication literacy of community-dwelling older adult patients with hypertension

Given the long-term nature of chronic diseases, improving medication literacy among community-dwelling older adult patients with hypertension deserves more focus. Enhancing medication literacy can lead to better disease management and improved quality of life in their later years. First, recognize the essential role of the external environment, such as the support from patients’ family members, friends, and medical staff, in improving medication literacy for community-dwelling older adult patients with hypertension. Efforts should be made to enhance the medical staff’s professional quality and widen the scope, method, and target of education. Second, emphasize the need for older adult patients with hypertension to strengthen their medication knowledge learning. Furthermore, implement psychological care to divert patients’ attention and increase their confidence in disease treatment. Lastly, communities should play a crucial role in chronic disease management by disseminating drug knowledge, advocating safe and rational drug use, and caring for older adult chronic disease groups.

## Conclusion

This study examined factors influencing the medication literacy of community-dwelling older adult patients with hypertension and constructed a pertinent structural equation. These findings will serve as a theoretical foundation for nursing staff to develop intervention programs to enhance medication literacy among older adult patients with hypertension. In practice, nursing staff should carry out individualized interventions. Focus on the impact of disease perception, self-efficacy, and social support on medication literacy. Nursing staff can improve medication literacy by strengthening social support, alleviating negative emotions about illness, and improving self-efficacy.

## Limitations

This research was conducted in a single city in China, thus the generalization of results requires further validation. Future studies should increase the sample size and conduct multi-center cross-sectional investigations.

## Data Availability

The raw data supporting the conclusion of this article will be made available by the authors, without undue reservation.
